# Ultra-high resolution multimodal MRI densely labelled holistic structural brain atlas

**DOI:** 10.1038/s41598-026-40186-2

**Published:** 2026-02-17

**Authors:** José V. Manjón, Sergio Morell-Ortega, Marina Ruiz-Perez, Boris Mansencal, Edern Le Bot, Marien Gadea, Enrique Lanuza, Gwenaelle Catheline, Thomas Tourdias, Vincent Planche, Remi Giraud, Denis Rivière, Jean-Francois Mangin, Nicole Labra-Avila, Roberto Vivo-Hernando, Gregorio Rubio, Fernando Aparici-Robles, Maria de la Iglesia-Vaya, Pierrick Coupé

**Affiliations:** 1 Instituto de Aplicaciones de las Tecnologías de la Información y de las Comunicaciones Avanzadas (ITACA), Universitat Politècnica de València, Camino de Vera s/n, Valencia, 46022 Spain; 2 CNRS, Univ. Bordeaux, Bordeaux INP, LABRI, UMR5800, in2brain, Talence, F-33400 France; 3 Department of Psychobiology, Faculty of Psychology, Universitat de Valencia, Valencia, Spain; 4 Department of Cell Biology, University Valencia, Burjassot, 46100 Valencia Spain; 5 University Bordeaux, CNRS, EPHE, PSL, INCIA, UMR 5283, Bordeaux, F-33000 France; 6 Service de Neuroimagerie diagnostique et thérapeutique, CHU de Bordeaux, Bordeaux, F-33000 France; 7 Institut des Maladies Neurodégénératives, Univ. Bordeaux, CNRS, UMR 5293, Bordeaux, F-33000 France; 8 Univ. Bordeaux, CNRS, Bordeaux INP, IMS, UMR 5218, Talence, F-33400 France; 9 NeuroSpin, BAOBAB lab, CEA Saclay, Gif-sur-Yvette, France; 10 Instituto de Automática e Informática Industrial, Universitat Politècnica de València, Camino de Vera s/n, Valencia, 46022 Spain; 11 Departamento de matemática aplicada, Universitat Politècnica de València, Camino de Vera s/n, Valencia, 46022 Spain; 12 Área de Imagen Médica. Hospital Universitario y Politécnico La Fe, Valencia, Spain; 13 Unidad Mixta de Imagen Biomédica FISABIO-CIPF. Fundación para el Fomento de la Investigación Sanitario y Biomédica de la Comunidad Valenciana, Valencia, Spain; 14 CIBERSAM, ISC III, Av. Blasco Ibáñez 15, València, 46010 Spain

**Keywords:** Atlas, Multimodal, Holistic, Segmentation, Brain volume analysis, MRI, Computational biology and bioinformatics, Medical research, Neurology, Neuroscience

## Abstract

In this paper, we introduce a novel structural holistic Atlas (holiAtlas) of human brain anatomy based on multimodal and high-resolution MRI that covers several anatomical levels from the organ level to the substructure level, using a new protocol for dense labelling generated from the fusion of multiple local protocols at different scales. This atlas was constructed by averaging images and segmentations of 75 healthy subjects from the Human Connectome Project database. Specifically, 3T MR images of T1, T2 and WMn (White Matter nulled) contrasts at 0.125 mm^3^ resolution were selected for this project. The images of these 75 subjects were nonlinearly registered and averaged using symmetric group-wise normalisation to construct the atlas. At the finest level, the proposed atlas has 350 different labels derived from 7 distinct delineation protocols. These labels were grouped at multiple scales, offering a coherent and consistent holistic representation of the brain across different levels of detail. This multiscale and multimodal atlas can be used to develop new ultra-high-resolution segmentation methods, potentially improving the early detection of neurological disorders. We make it publicly available to the scientific community.

## Introduction

The field of neuroscientific research has been revolutionized by the development of advanced imaging techniques, with Magnetic Resonance Imaging (MRI) standing at the forefront. MRI provides a non-invasive and high-resolution approach to investigate the details of human brain anatomy. In particular, the development and utilization of MRI-based brain atlases have emerged as valuable tools that play an important role to standardize image resolutions, orientations and label definitions, and to provide a common ground for brain research worldwide which has definitely helped in understanding the complex architecture of the brain.

A brain atlas serves as a reference framework that maps and delineates various anatomical and functional regions within the brain. It provides a standardized coordinate system, allowing researchers to precisely locate regions of interest at the individual level and also compare them across individuals or populations. The importance of MRI-based brain atlases lies in their multifaceted utility across diverse domains of neuroscientific investigation.

MRI-based brain atlases enable detailed structural mapping of the brain, allowing researchers to obtain anatomical references to study the morphology of different regions. This aids in the identification of anatomical landmarks and exploring the spatial relationships between brain structures. In the clinical realm, MRI-based brain atlases are indispensable tools for accurate diagnosis and treatment planning. Neurosurgeons rely on specialized surgical software using these atlases to navigate the brain during surgical procedures, ensuring precision in targeting specific areas while minimizing damage to surrounding healthy tissue. Brain atlases also serve as a common reference for population-based studies^1^, allowing researchers to compare and contrast brain structures across diverse demographic groups to better understand the natural history of neurological diseases^2–4^. They facilitate the integration of data from multiple studies, fostering collaborative efforts and meta-analyses to derive more robust conclusions.

Several brain atlases have been developed over the years, each serving distinct purposes in neuroscientific research, clinical applications, and medical practice. The Talairach and Tournoux Atlas^5^, originally developed for stereotactic neurosurgery, introduced a three-dimensional grid system based on post-mortem anatomical landmarks, serving as an early milestone for spatial normalization. However, modern neuroimaging requires more sophisticated and standardized approaches. The Montreal Neurological Institute (MNI) Atlas (MNI305)^1^ addressed this need by offering a standardized coordinate system, facilitating meta-analyses and cross-study comparisons (in 2001 an improved version of this atlas named MNI152^6–8^ was proposed and is currently one of the most used ones)^9^. proposed an atlas with 49 labelled structures^10^. also proposed a probabilistic atlas of human cortical structures. Later, with a larger number of structures (*n* = 90), the AAL (Automated Anatomical Labeling) Atlas^11–13^ was proposed and has been widely used in many functional neuroimaging studies, providing automated labelling of brain regions for voxel-based analyses. More recently, the Brainnetome Atlas^14^, was designed to help in the analysis of brain networks and connectivity, providing insights into functional and structural connections between different brain regions. From a more structural perspective, the Desikan-Killiany Atlas^15^ and the MINDboggle Atlas^16^ have also been commonly used in structural MRI studies.

There are also brain atlases derived from histology that have exceptional detail. The Allen Human Brain Atlas^17^ (https://atlas.brain-map.org/) combines different types of data, including anatomical reference maps based on Nissl-stained sections, gene expression data from microarray and in situ hybridization (ISH) studies, and MRI scans. The Allen Atlas is crucial for understanding the regional molecular organization of different brain areas and includes detailed maps of gene expression across the entire human brain, allowing researchers to explore the genetic basis of brain function. BigBrain template^18^ is a model of a human brain at a cellular resolution of 20 micrometers, based on the reconstruction of 7404 histological sections of a single brain. More recently, a probabilistic atlas, named Julich-Brain^19^ was proposed. However, these atlases are not MRI-based and their use with clinical-quality MRI data can be challenging.

All these atlases, among others, serve as essential tools in neuroscientific research, providing a standardized framework for the interpretation and comparison of brain imaging data. Their diverse features meet several research needs, from structural and functional mapping to gene expression patterns and connectivity analysis. An extensive list of many of them can be found here (https://fsl.fmrib.ox.ac.uk/fsl/fslwiki/Atlases).

Currently, the MRI-based atlases have a maximum resolution of 1 mm^3^ and typically use the T1 weighted (T1w) image modality due to its high anatomical contrast. However, higher image resolutions are starting to be available^20,21^ thanks to either the use of new ultra-fast MR acquisitions (many of them powered nowadays by the use of artificial intelligence^22^) or by the use of post-acquisition super-resolution techniques^23,24^. Initiatives to produce higher-resolution atlases are currently in development^25,26^. Also, multimodal approaches have been explored^27^.

In this paper, we propose a new structural holistic MRI-based ultra-high resolution multimodal densely labelled atlas. This atlas is based on ultra-high resolution in vivo MRI and has been labelled using the fusion of currently available tools for brain parcellation. The improved resolution of the atlas (0.125 mm^3^ vs. typical 1 mm^3^), its multimodal nature and its dense and holistic labelling will facilitate the measurement of more subtle anatomical patterns and hopefully will contribute to earlier diagnostics and analyses. In the next sections, the atlas construction details and the resulting atlas are described.

## Materials and methods

To construct the aforementioned structural atlas, we used images from a public dataset and a private dataset, and segmentations from existing tools as a starting point. In this section, the details of this process are summarized.

### Dataset description

We used MR images from 2 datasets to construct the atlas. On the one hand, we randomly selected 75 healthy subjects (T1w and T2w paired images) from the Human Connectome project (HCP), specifically, the HCP1200 dataset (https://www.humanconnectome.org/study/hcp-young-adult/document/1200-subjects-data-release). Those images were taken in a 3T MR scanner from healthy subjects (41 females and 34 males) with ages between 22 and 35 years. High-resolution T1w and T2w images had a matrix size of 260 × 311 × 260 voxels and a voxel size of 0.7 × 0.7 × 0.7 mm^3^. On the other hand, we used 55 young healthy voluntary subjects from a private dataset acquired on a 3T scanner (Vantage Galan 3T/ZGO; Canon Medical Systems) at Bordeaux Hospital as part of the DeepMultiBrain research project. In this dataset, each subject had T1w, T2w and WMn (White Matter nulled) images. In this dataset, T1w and T2w images had a matrix size of 256 × 376 × 368 voxels and a voxel size of 0.6 × 0.6 × 0.6 mm^3^. WMn images had a matrix size of 448 × 548 × 400 voxels and a voxel size of 0.4 × 0.4 × 0.4 mm^3^. WMn images have an excellent contrast for deep gray matter structures, especially useful for thalamic nuclei segmentation. This dataset was not used in the atlas construction but to generate synthetic WMn not available in the HCP dataset.

### Image preprocessing

All selected T1w and T2w images from the HCP1200 dataset underwent multi-step preprocessing to stage to place them in a standard intensity and geometric space. First, both images were denoised using the Spatially Adaptive Non-local means (SANLM) filter^28^ and later inhomogeneity corrected using the N4 bias correction method^29^. The filtered images were then affine-registered to the MNI152 space at 0.125 mm^3^ resolution (voxel size of 0.5 × 0.5 × 0.5 mm^3^) using ANTs software^30^. The resulting images have a standard matrix size of 362 × 434 × 362 voxels. Finally, the images were intensity normalized using the TMS method^31^.

For the DeepMultiBrain Bordeaux dataset, the same preprocessing was applied with the exception that rigid transformation from native T2w and WMn images to the native T1w was first estimated to later concatenate it with the affine transform of T1w image to map all the images to the same MNI152 space (HCP T1w and T2w images were already registered).

**Fig. 1:**
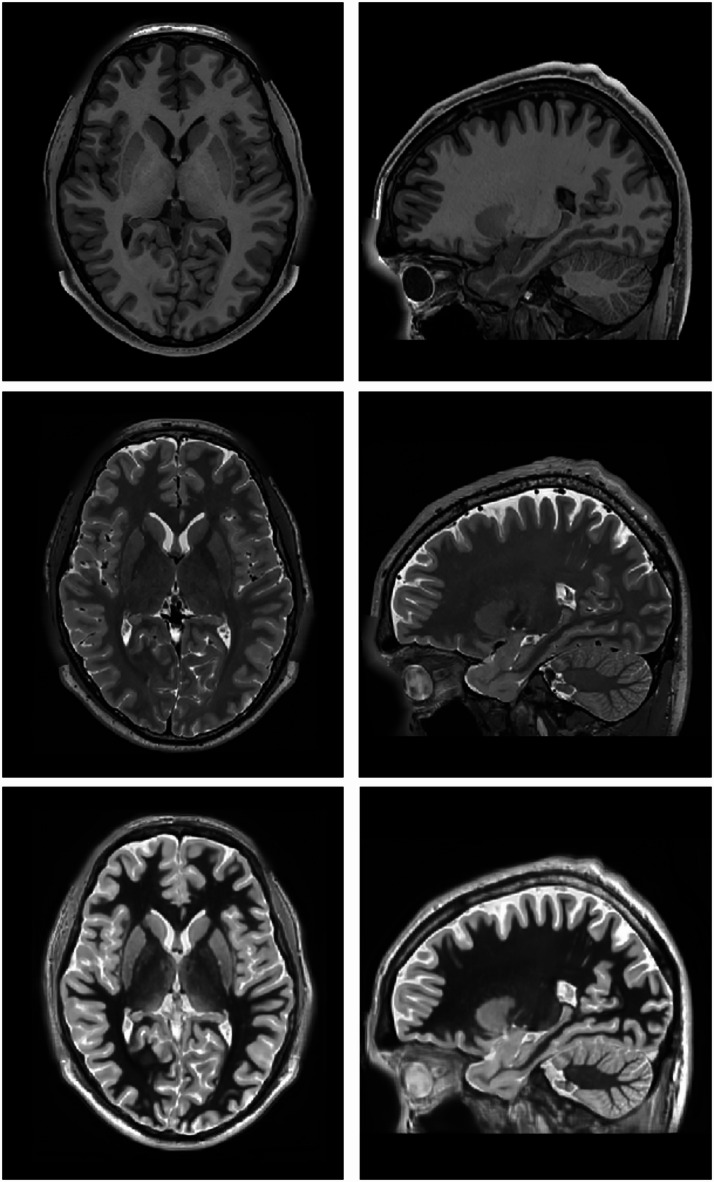
Example of a preprocessed T1w, T2w and the synthetic WMn images at 0.125 mm^3^ resolution MNI152 space.

As we wanted to use public data in the creation of the atlas, we decided to synthetize the WMn images using HCP1200 data instead of using the DeepMultiBrain Bordeaux dataset. Modern image synthesis techniques now enable the generation of non-acquired modalities from existing ones^32,33^. To generate WMn-like images in the HCP1200 dataset, a multimodal variant of a full-volume synthesis method^33^ was used. We trained this variant using the DeepMultiBrain Bordeaux dataset where the input images (T1w and T2w) were used to generate a WMn image. Once the network was trained, it was applied to the HCP dataset to generate the WMn images. Since this network works at 1 mm^3^ resolution, it was applied 8 times to a volume-to-channel decomposition that transforms the input volume of 362 × 548 × 362 voxels into 8 volumes of 181 × 217 × 181 voxels using striding with step 2 at each dimension as done in method DeepICE^34^. After synthetizing the 8 WMn volumes the process is reversed to obtain the final 362 × 548 × 362 voxel WMn volume. Figure 1 shows an example of the T1w, T2w and synthetized WMn images. The quality of the synthetized WMn images was qualitatively validated by our experts (MG and TT).

### Software packages

To generate a densely labelled atlas, we decided to merge several available protocols from 7 different software packages. These software packages were used to automatically segment the 75 HCP subjects used to build the atlas. Those automatic segmentation were later semi-automatically corrected and fused (and also manually corrected when needed). The seven software packages used are the following:

1. **vol2Brain**^35^: This software is able to segment the brain into 135 regions. It is based on non-local multi-atlas label fusion and is available as an online tool at https://volbrain.net. We selected vol2Brain because it is a state-of-the-art method for full brain segmentation. vol2Brain software was validated by the authors giving an average dice coefficient of 0.8262 (135 regions).
2. **hypothalamus_seg**^36^: This method is able to segment the hypothalamus into different parts using a convolutional neural network. The code is available on GitHub (https://github.com/BBillot/hypothalamus_seg). We selected this software because it is, as far as we know, the only method available for hypothalamus nuclei segmentation. Its average dice was estimated to be 0.84 from the authors’ measurements on an external dataset.
3. **BrainVISA**^37^: BrainVisa is a software for brain segmentation and sulcal morphometry and was used to segment the brain sulci. The software is available at https://brainvisa.info. This software has several updates in recent years^38,39^. We selected Brainvisa because it is considered the reference method for sulci estimation.
4. **Freesurfer**^40^: Freesurfer is a well-known software package to segment the brain and analyze cortical thickness. The software can be downloaded here: https://surfer.nmr.mgh.harvard.edu. In the construction of the atlas, we used several subregion segmentation tools integrated into Freesurfer 7.3. Specifically, we used the tools for brainstem^41^ and amygdala^42^/hippocampus^43^ segmentation. We used Freesurfer as it is also a standard method in brain segmentation including brainstem and amygdala/hippocampus subparts.
5. **pBrain**^44^: This software is able to segment the 3 deep grey matter structures related to Parkinsonism (substantia nigra, red nucleus and subthalamic nucleus). It is based on non-local multi-atlas label fusion and is available as an online tool at https://volbrain.net. pBrain is the only method available for segmenting these deep gray matter structures. The average dice reported by the authors is 0.89.
6. **HIPS**^45^: This software is able to segment the hippocampus subfields with 2 different delineation protocols (with 3 and 5 labels each). It is also based on non-local multi-atlas label fusion and is available as an online tool at https://volbrain.net. We selected HIPS because our experts preferred the Winterburn delineation protocol (5 labels) compared to Freesurfer´s one (especially the inclusion of the tail label). We actually combined both protocols as we will describe later.
7. **CERES**^46^: CERES is an online automated software to segment the cerebellum lobules. It represents the current state of the art on cerebellum segmentation and is available as an online tool at https://volbrain.net. We selected CERES because it represented the state of the art for cerebellum segmentation at the time of atlas creation (it won the MICCAI challenge for cerebellum segmentation^47^).

### Protocol integration process

To fully label each subject, we ran the described software and adapted the results to incrementally label them. The first applied pipeline was the vol2Brain software. This software segments a T1w image into 135 cortical and subcortical labels^35^. Because this method works at 1 mm^3^ resolution and not at 0.125 mm^3^ resolution, we decomposed the high-resolution T1w volume of size 362 × 434 × 362 voxels into 8 volumes of 181 × 217 × 181 voxels using a stride decomposition (step = 2 in each dimension)^34^. The eight volumes were segmented using vol2Brain and the resulting segmentations were composed back to the high-resolution space by inverting the striding operation over the label maps. This resulted in a single volume of size 362 × 434 × 362 fully labelled with 135 labels (we used this approach because it gave much better results than labelling at 1 mm^3^ and later interpolating to 0.125 mm^3^ resolution).

#### Tissue error correction

**Fig. 2:**
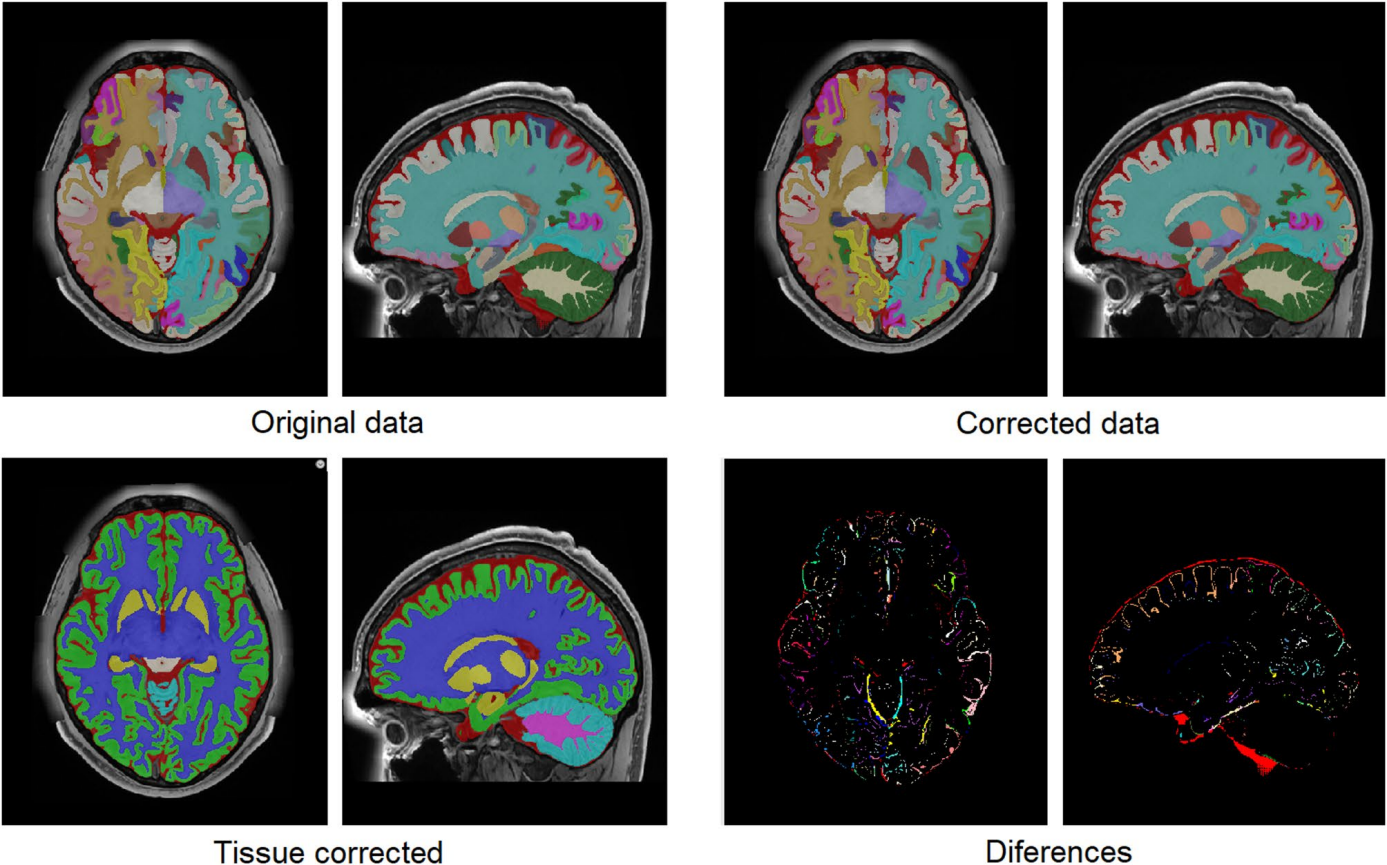
Top-Left: Original segmentation before correction. Bottom-Left: Corrected tissue segmentation. Top-Right: Corrected segmentation using the corrected tissue maps. Bottom-Right: Difference between the original and corrected segmentation.

After this automatic segmentation, some systematic errors were present. Mainly, dura and vessels were misclassified as grey matter and sulcal CSF was underestimated. As these errors were found at the tissue level, the structure segmentation was automatically relabeled from 135 labels to 7 tissues (CSF, cortical GM, cerebral WM, deep GM, cerebellum GM, cerebellum WM and brainstem). To enforce the regularity of the different tissues, their probability maps were filtered using a non-local means filter using as reference the T1w and T2w intensities to estimate the voxel similarities (this largely improved CSF underestimation). To correct GM misclassification, we trained a UNET-like deep convolutional network using 12 cases manually corrected (this manual correction was performed using ITK-SNAP^48^). The trained network was used to correct the remaining 63 subjects. Finally, the 75 cases were visually checked and the remaining errors were manually corrected. Once the tissues were corrected, we used these maps to automatically correct the original structure segmentation. To do so, a spatial diffusion process was used. In this process, all voxels that did not change their tissue class were preserved but the remaining ones were relabelled. To perform this relabelling we used the spatial and intensity proximity to assign the new labels. Specifically, the probability map of each structure was smoothed using a 3D Gaussian kernel (to compute a spatial a priori probability map) and this information jointly with the voxel intensity was used to assign the new label. Basically, we used an iterative Bayesian Maximum A Posteriori (MAP) approach where the a priori probability was in the form of a spatial probability map, and the likelihood of the intensities was calculated as the value of a Gaussian distribution at a given intensity with respect to the mean of the neighboring structures normalized by the variance of the structure intensities. An example result can be seen in Fig. 2. This process was supervised by JVM and PC.

#### Hypothalamus integration

The second protocol to integrate was the hypothalamus one. Hypothalamus_seg software segments the hypothalamus into 10 substructures. These labels were mapped on top of left and right white matter and partial volume voxels misclassified as WM in the border of hypothalamus/CSF were reclassified using the described spatial/intensity diffusion process. The added labels of the hypothalamus were: Right Anterior Inferior Hypothalamus, Left Anterior Inferior Hypothalamus, Right Anterior Superior Hypothalamus, Left Anterior Superior Hypothalamus, Right Posterior Hypothalamus, Left Posterior Hypothalamus, Right Tubular Inferior Hypothalamus, Left Tubular Inferior Hypothalamus, Right Tubular Superior Hypothalamus and Left Tubular Superior Hypothalamus. This process was supervised by JVM and PC. While these subdivisions do not correspond one-to-one with histological nuclei, they approximately map onto known functional territories. *Anterior–Superior* includes regions containing the preoptic area and paraventricular nucleus, involved in thermoregulation, reproductive behaviors, neuroendocrine regulation (including stress responses) and osmoregulation, *Anterior–Inferior* encompasses the supraoptic and suprachiasmatic nuclei, involved in osmoregulation and circadian control, respectively, *Tubular–Superior* overlaps with dorsomedial nucleus and lateral hypothalamus, associated with feeding behavior, *Tubular–Inferior* includes the arcuate nucleus, involved in metabolic and hormonal regulation and the ventromedial nucleus; lateral tubular nucleus and tuberomammillary nucleus and *Posterior* covers the posterior hypothalamic and mammillary regions, related to memory, arousal and autonomic functions.

#### Sulci integration

The cortical sulci were obtained from the T1w images via the following steps embedded into the Morphologist pipeline of BrainVISA (http://brainvisa.info^37^), . First, the brain mask was obtained with an automated skull stripping procedure including bias correction, histogram scale-space analysis and mathematical morphology. Second, the brain mask was split into hemispheres and segmented into grey matter, white matter and cerebrospinal fluid. Third, a negative cast of the cortical folds was segmented and labelled into sulci. The fold segmentation results from a 3D crevasse detector reconstructing each fold geometry as the medial surface from the two opposing gyral banks using a watershed procedure. A Bayesian pattern recognition approach relying on Statistical Probabilistic Anatomy Maps and multiscale spatial normalization was used to label the folds using a nomenclature of 124 sulci^38^.

**Fig. 3:**
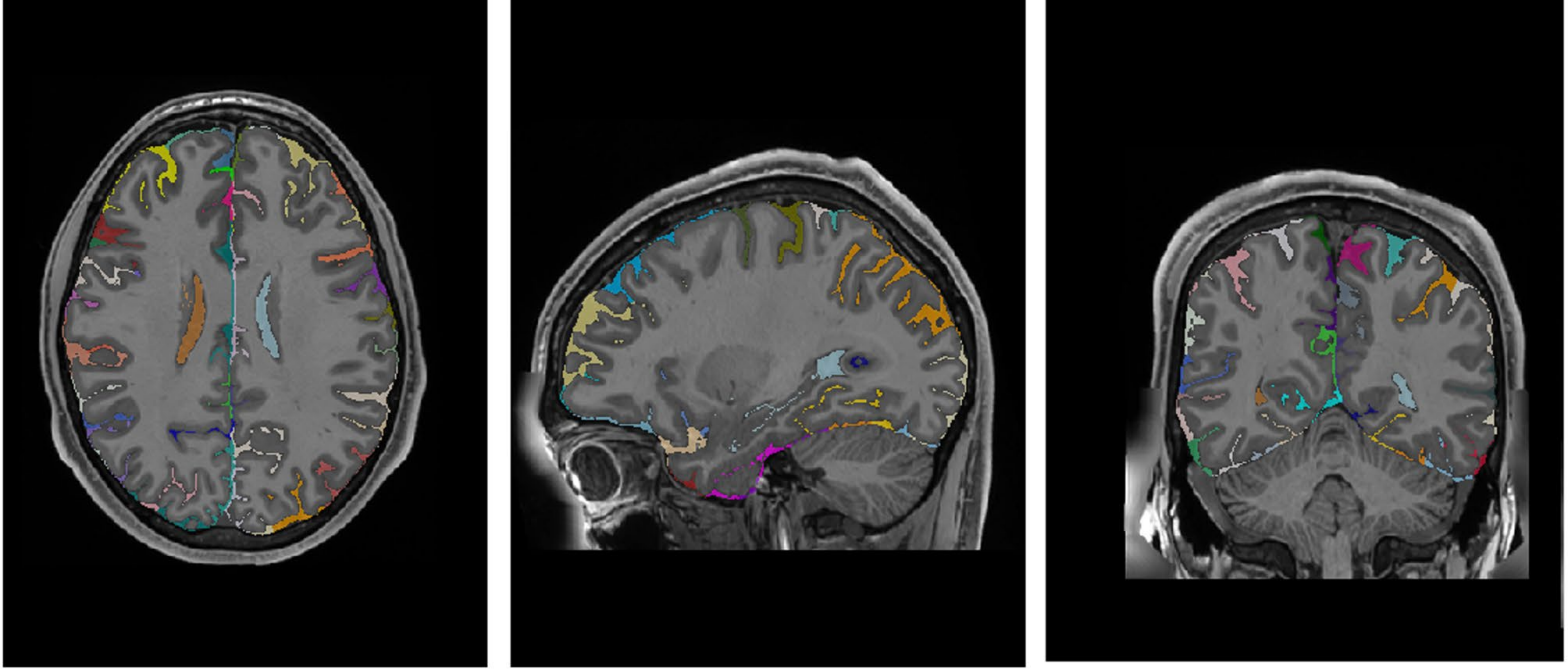
Example of 3D sulci segmentation based on 2D mesh BrainVISA sulci labeling.

To integrate this sulcus information, each external CSF voxel (label 8) was relabeled according to the most proximal sulcus using the automatic sulci segmentation (see Fig. 3). After mapping the sulcal labels into label 8, the CSF not labelled in the proximity of sulcal labels was relabeled using the spatial-intensity diffusion process. The remaining external CSF was left unchanged (label 8) (mainly cerebellar and brainstem CSF and areas of the top of the brain far from the sulci). After the automatic segmentation, visual QC was performed, and small remaining errors were manually corrected using ITK-SNAP (mainly CSF voxels close to the cerebellum and brainstem). This process was supervised by EL, JFM and DR. In Fig. 3 an example case of the labelling is shown.

#### Brainstem and amygdala integration

The next two structures to be integrated were obtained using Freesurfer: brainstem and amygdala. Again, new labels for brainstem and amygdala were transferred to the new protocol and old labels for brainstem and amygdala were reclassified based on the bilateral spatial and intensity probabilities using the described MAP-based approach.

**Fig. 4:**
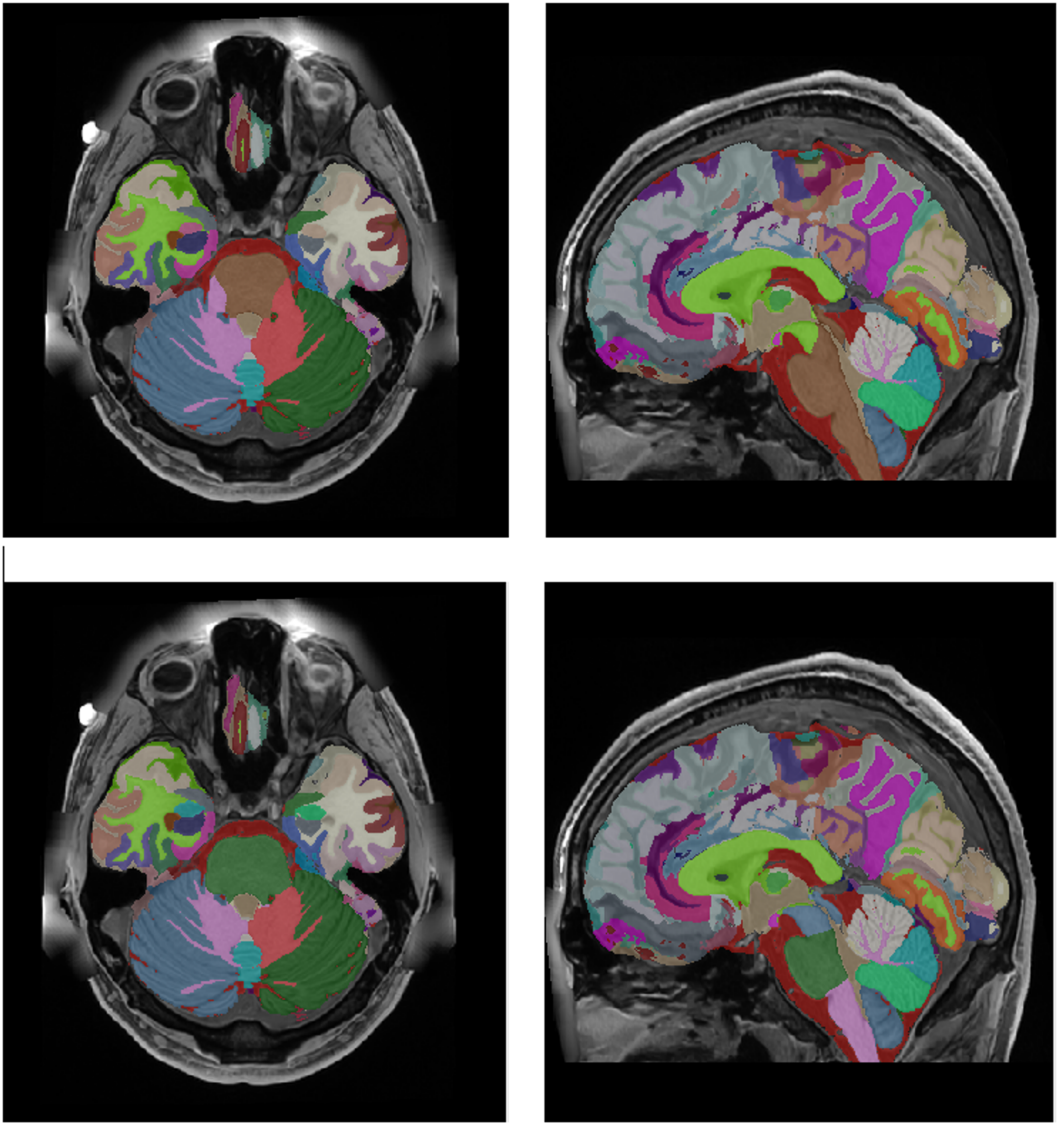
Top row: original labels. Bottom row: integrated new labels. Note that the brainstem is now divided in its constituent parts.

The Brainstem was divided into 4 regions: Midbrain, Pons, Medulla and Superior Cerebellar Peduncle. The new definition of the brainstem was slightly bigger than the vol2brain definition and overwrote some WM areas. Partial volume voxels were reclassified as brainstem or CSF again according to their intensity and location. Each amygdala was divided into 8 regions: Lateral Nucleus, Basal Nucleus, Central Nucleus, Medial Nucleus, Cortical Nucleus, Accessorial Nucleus, Cortico-Amygdaloid Transition and Paralaminar Nucleus. The new amygdala was also bigger than the vol2brain definition. Anterior Nucleus was not included in its definition as it was really small and occupied part of the entorhinal area. The amygdala segmentation had some systematic errors overestimating the frontal and inferior parts. In the Brainstem, the superior cerebellar peduncle was commonly overestimated and the medulla was underestimated in its inferior part. Manual corrections were performed when needed to correct these errors using ITK-SNAP. This process was supervised by MG and EL. Figure 4 shows an example of the relabeling process for the brainstem.

#### pBrain structures integration

**Fig. 5:**
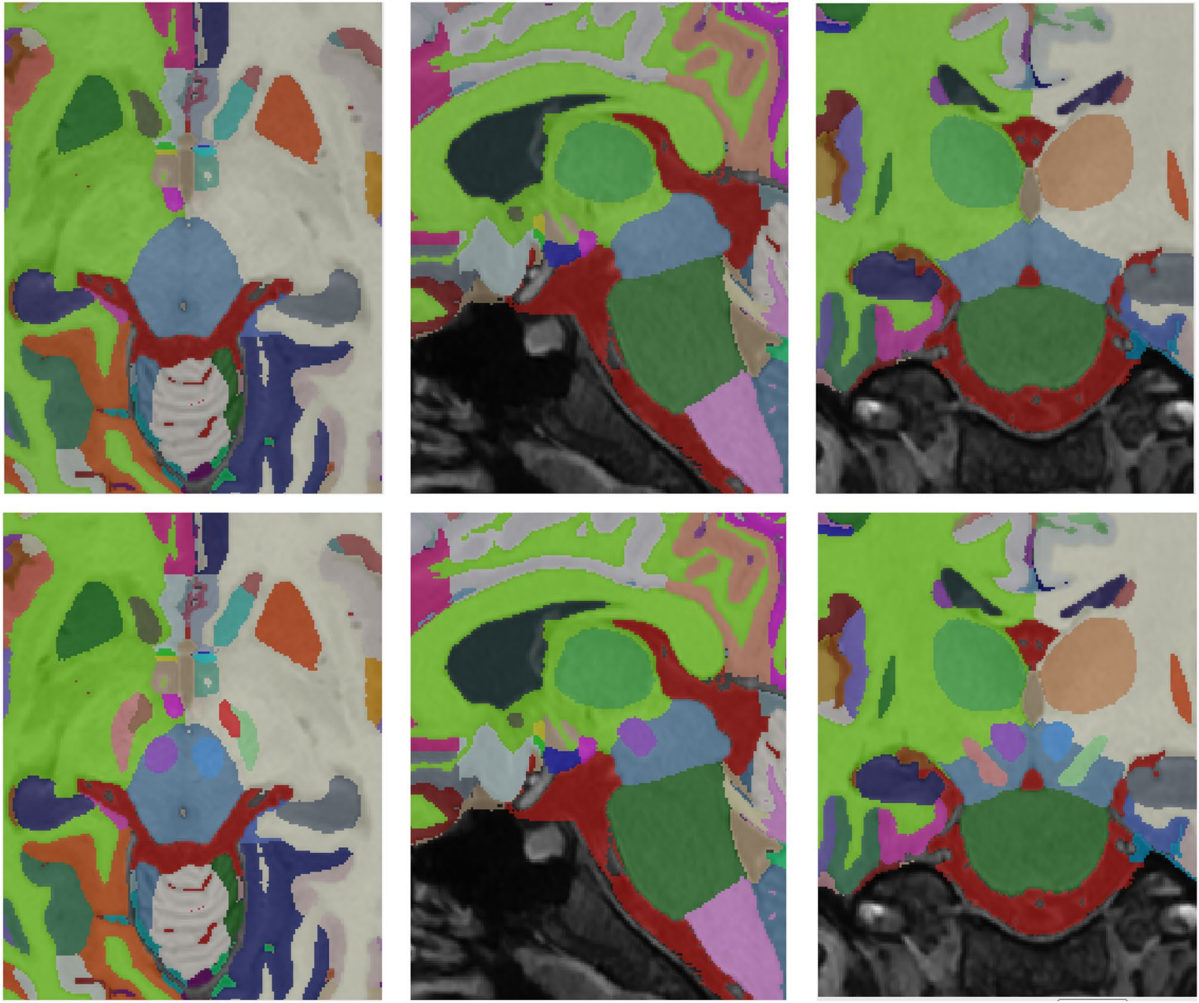
Top row: original labels. Bottom row: integrated new subthalamic nuclei labels. Note that new labels modify the brainstem labels.

The pBrain pipeline provides segmentation of 3 structures of deep grey matter per hemisphere (red nucleus, substantia nigra and subthalamic nucleus) using a HR T2w image. As in the case of hypothalamus segmentation, labels were simply transferred to the corresponding white matter and midbrain region. The main problem with this integration was the fact that these structures partially occupied part of the midbrain (see Fig. 5). Although these structures could be artificially divided to consider this fact, we decided for simplicity to define a *partial* midbrain as a result of this integration process which was supervised by JVM and EL.

#### Hippocampus subfield integration

**Fig. 6:**
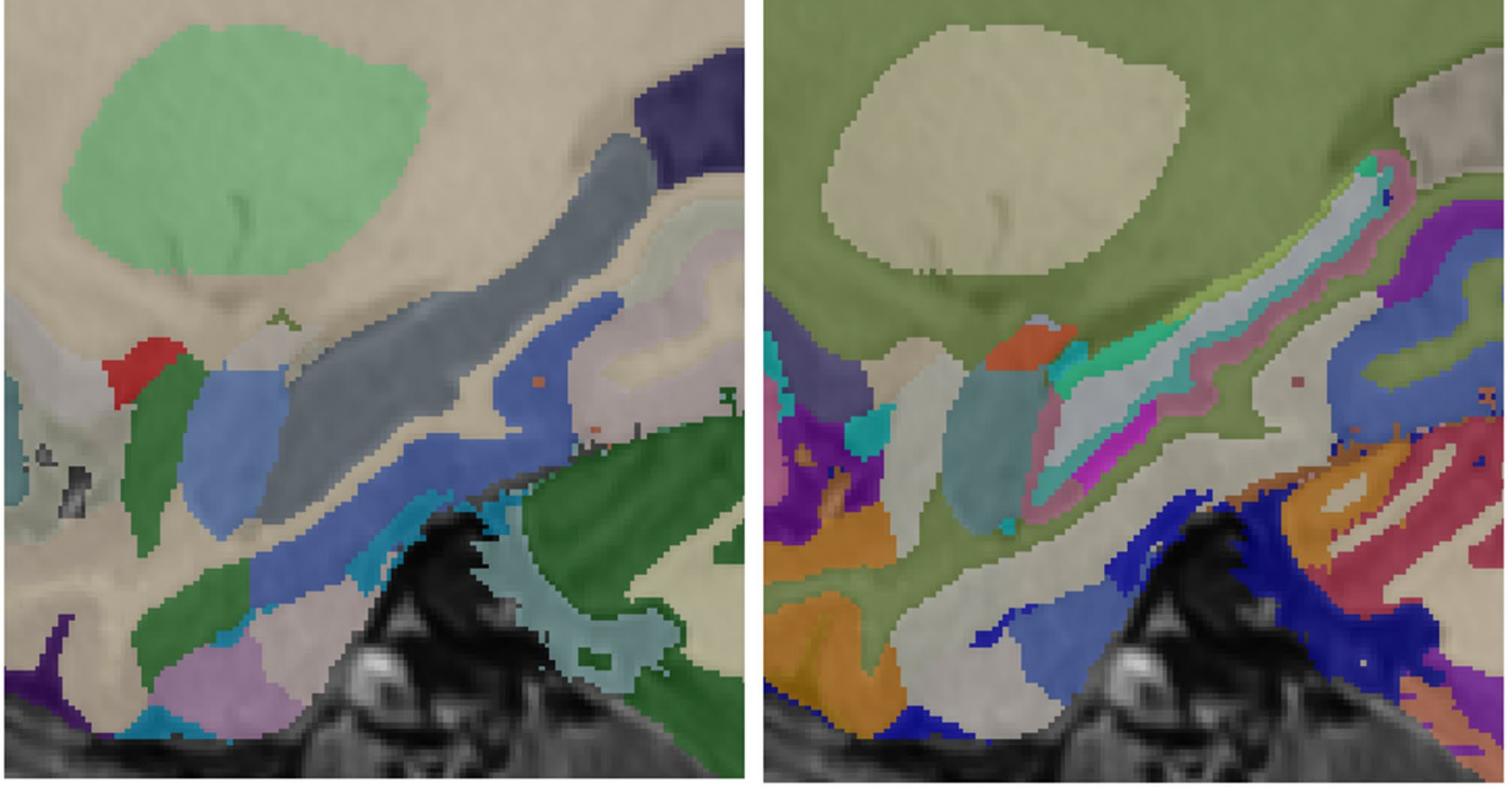
Example of the old and new hippocampus subfield definition on top of a reference T1w MRI.

To label the hippocampus subfields, we used the software HIPS^45^. HIPS segmented the hippocampus into 5 subfields (CA1, CA2/3, CA4/DG, SR/SL/SM and Subiculum) using the Winterburn protocol^61^. However, we added two new structures, the Fimbria and the Hippocampal-Amygdalar Transition Area (HATA) to connect the hippocampus head with the amygdala. We obtained these labels from Freesurfer segmentation and fused with HIPS outputs. CSF pockets were classified as CSF. To map the new hippocampus definition over the vol2Brain-defined hippocampal area, first the vol2Brain hippocampus label was reclassified as WM and later the new definition was transferred only in the destination voxel has the WM label (to avoid amygdala label modifications). A throughout QC was done using ITK-SNAP and manual corrections were applied when necessary. The responsible to supervise the hippocampus integration were VP and EL. Figure 6 shows an example of the extended hippocampal protocol.

#### Thalamus subfield integration

**Fig. 7:**
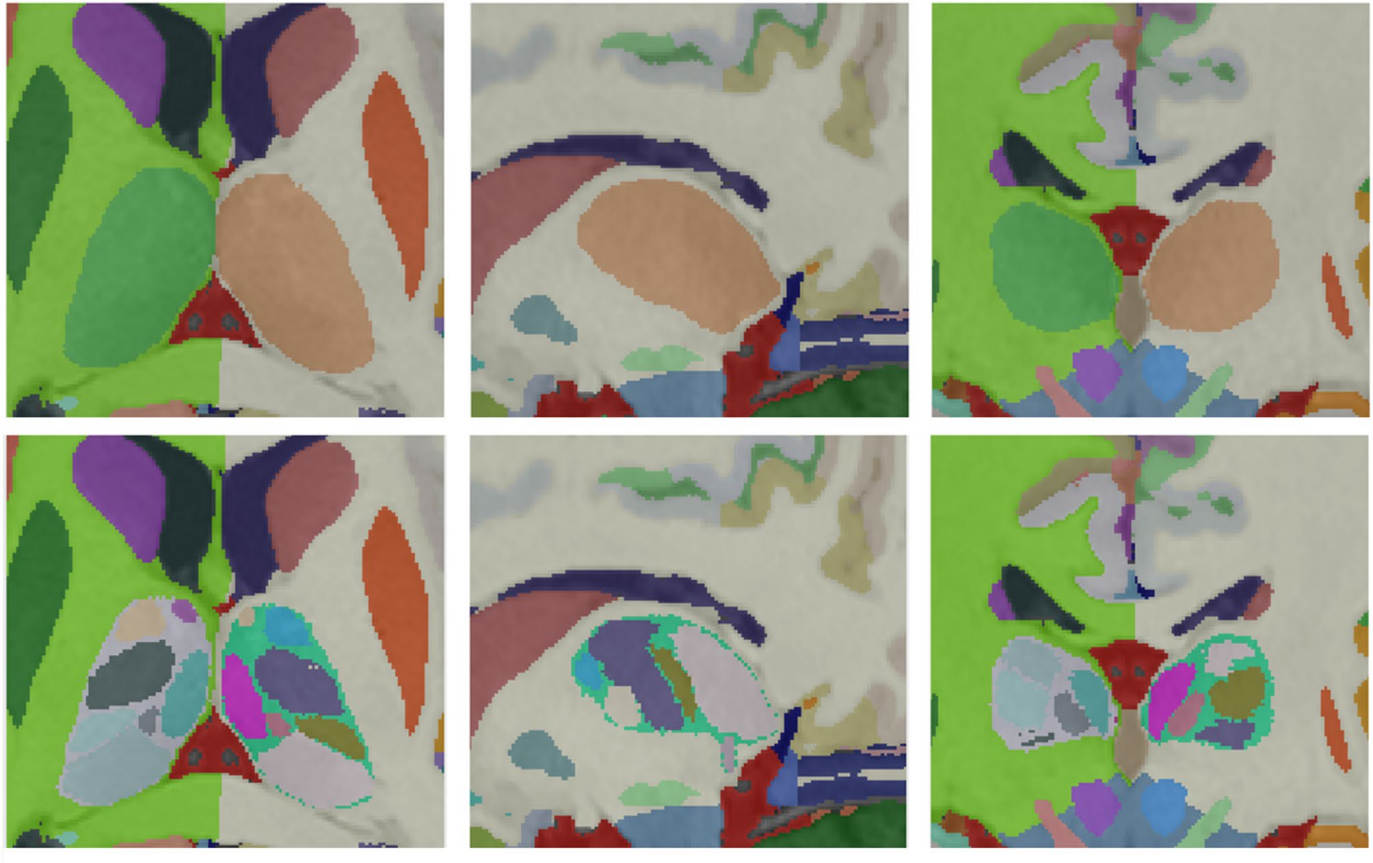
Top row: original labels. Bottom row: integrated new thalamic nuclei labels.

**Fig. 8:**
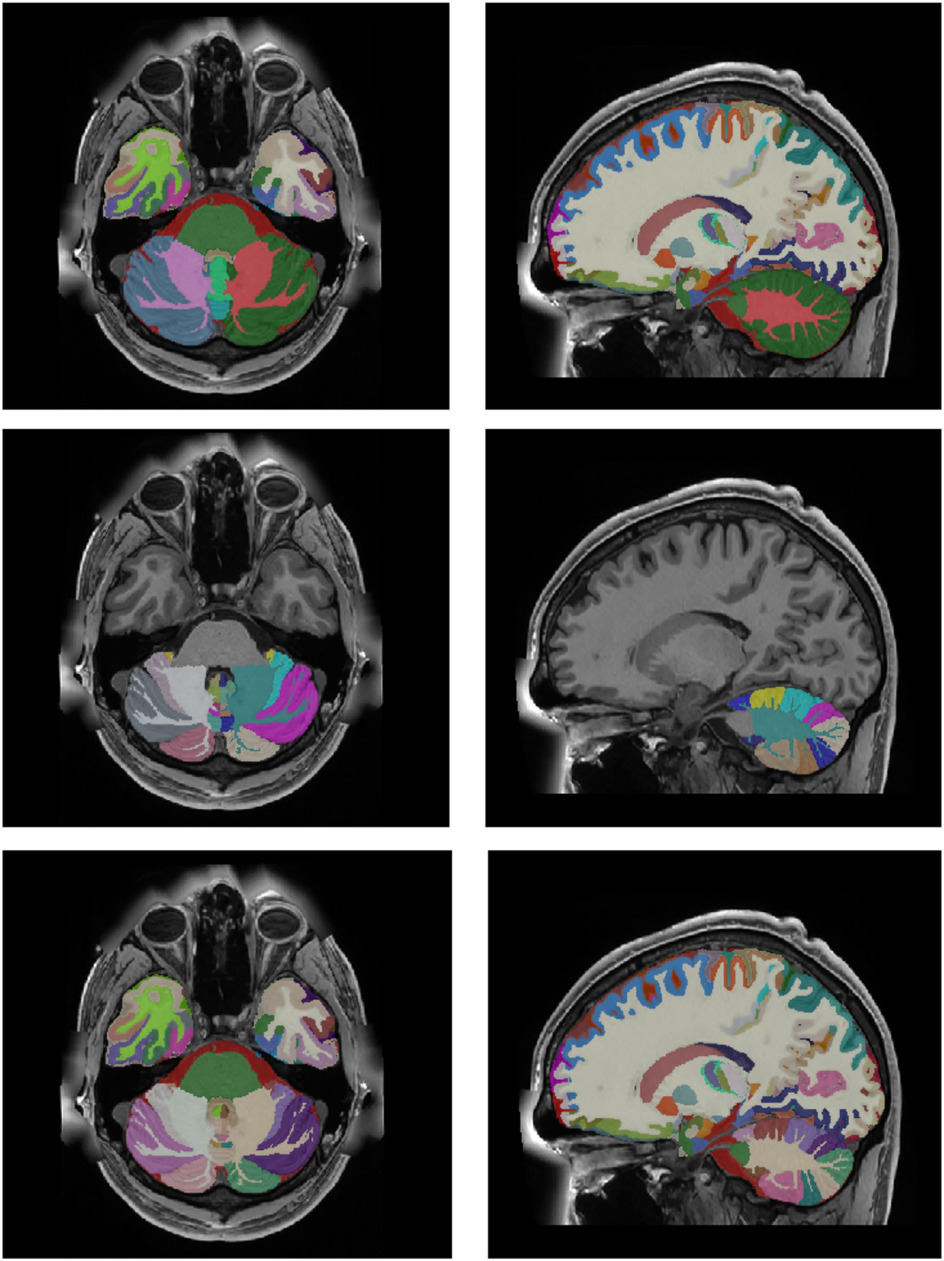
Top row: original labels. Middle row: CERES segmentation. Bottom row: integrated new cerebellum labels.

To obtain the thalamus nuclei we used the THOMAS software training dataset (https://github.com/thalamicseg/thomas_new). This dataset contains 20 White Matter Nulled (WMn) subjects. Specifically, we trained a deep neural network to segment WMn images and we used it to segment the 75 WMn images of our dataset. We used this network instead of using the THOMAS software because it provided better segmentations than THOMAS thus requiring less manual editing. The Thomas protocol provides segmentation of 12 nuclei of the thalamus (Anterior Ventral Nucleus, Ventral Anterior Nucleus, Ventral Lateral Anterior Nucleus, Ventral Lateral Posterior Nucleus, Ventral Posterior Lateral Nucleus, Pulvinar Nucleus, Lateral Geniculate Nucleus, Medial Geniculate Nucleus, Centromedian Nucleus, Mediodorsal Nucleus, Habenular Nucleus and Mammillothalamic Tract). To label the entire thalamus as a whole we added a new label named *intermediate space*, defined as the WM region connecting all the nuclei, resulting in 13 total labels per thalamus (see Fig. 7). In this case, the new thalamus definition was slightly smaller than the vol2Brain definition. This process was supervised by MR, MG and TT. Again, the QC was performed using ITK-SNAP and manual correction was applied when needed (specifically, main errors were found at Mammillothalamic Tract and Habenular Nucleus which are the smaller nuclei) (Fig. 8).

#### Cerebellum lobules integration

**Fig. 9:**
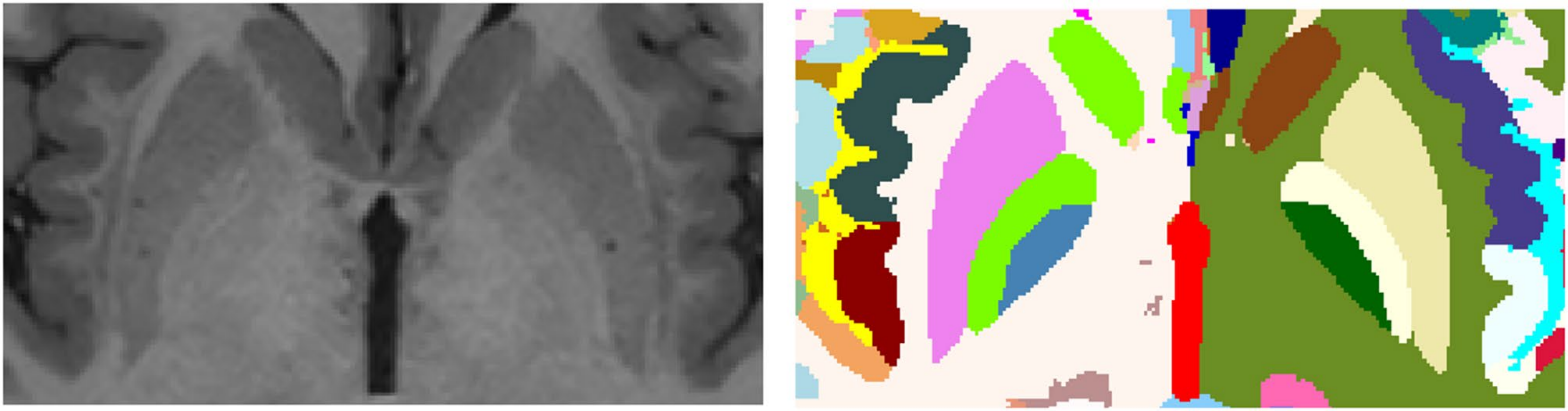
Left: T1w image. Right: Pallidum segmentation of their internal and external parts.

To segment the cerebellum, we used the CERES method^46^, a multi-atlas path-based label fusion technique. CERES provides segmentation of 12 lobules of the cerebellum (Lobule I-II, Lobule III, Lobule IV Lobule V, Lobule VI, Lobule Crus I, Lobule Crus II, Lobule VIIB, Lobule VIIIA, Lobule VIIIB, Lobule IX and Lobule X) plus the cerebellar white matter (see Fig. 9). CERES works at 1 mm^3^ resolution. Therefore, to generate the segmentation at 0.125 mm^3^ resolution, we used the same striding-based approach we used previously. The new cerebellum definition is slightly smaller than the previous vol2Brain one, mainly due to WM label. We decided to increase the WM label combining both. The non-overlapping area was reclassified based on spatial and intensity information. Finally, the increased resolution of the images allowed to properly segment the intra-lobular white matter (not visible at 1 mm^3^ resolution due to partial volume effects). This was done by SM as described in DeepCERES method^49^. Figure 8 shows an example of this integration process.

#### Pallidum integration

Finally, we decided to manually segment one structure for deep grey matter completeness. We divided the globus pallidus into its internal and external parts manually. This was done using ITK-SNAP from the entire pallidum segmentation obtained with vol2Brain pipeline. Figure 9 shows and example of the pallidum parcellation. This was done by JVM under the supervision of FA and MG.

#### Vessels and connective tissue

**Fig. 10:**
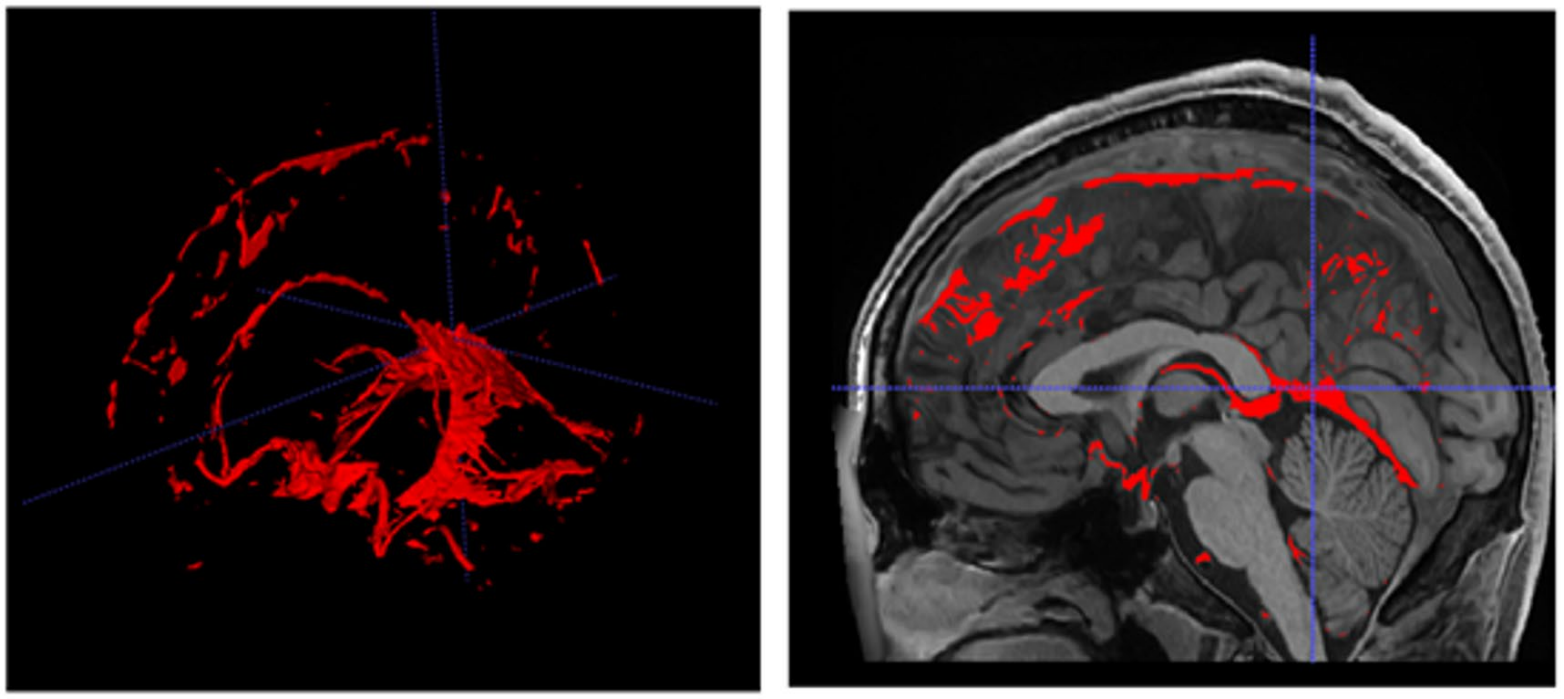
Right: Example of Vessels and connective tissue. Left: 3D representation of this label.

After the whole integration process, we automatically performed a 3D hole-filling operation over the binary mask formed by the sum of all the defined structures (i.e. the foreground) to get a solid intracranial cavity volume (no holes). We named the label corresponding to the filled regions “vessels+ connective tissue”, as this label is mainly related to these tissues. Figure 10 shows an example of this label. This label entails a more compact brain anatomy and to fully define all the structures with the intracranial volume (ICV).

#### Multiscale label generation

**Fig. 11:**
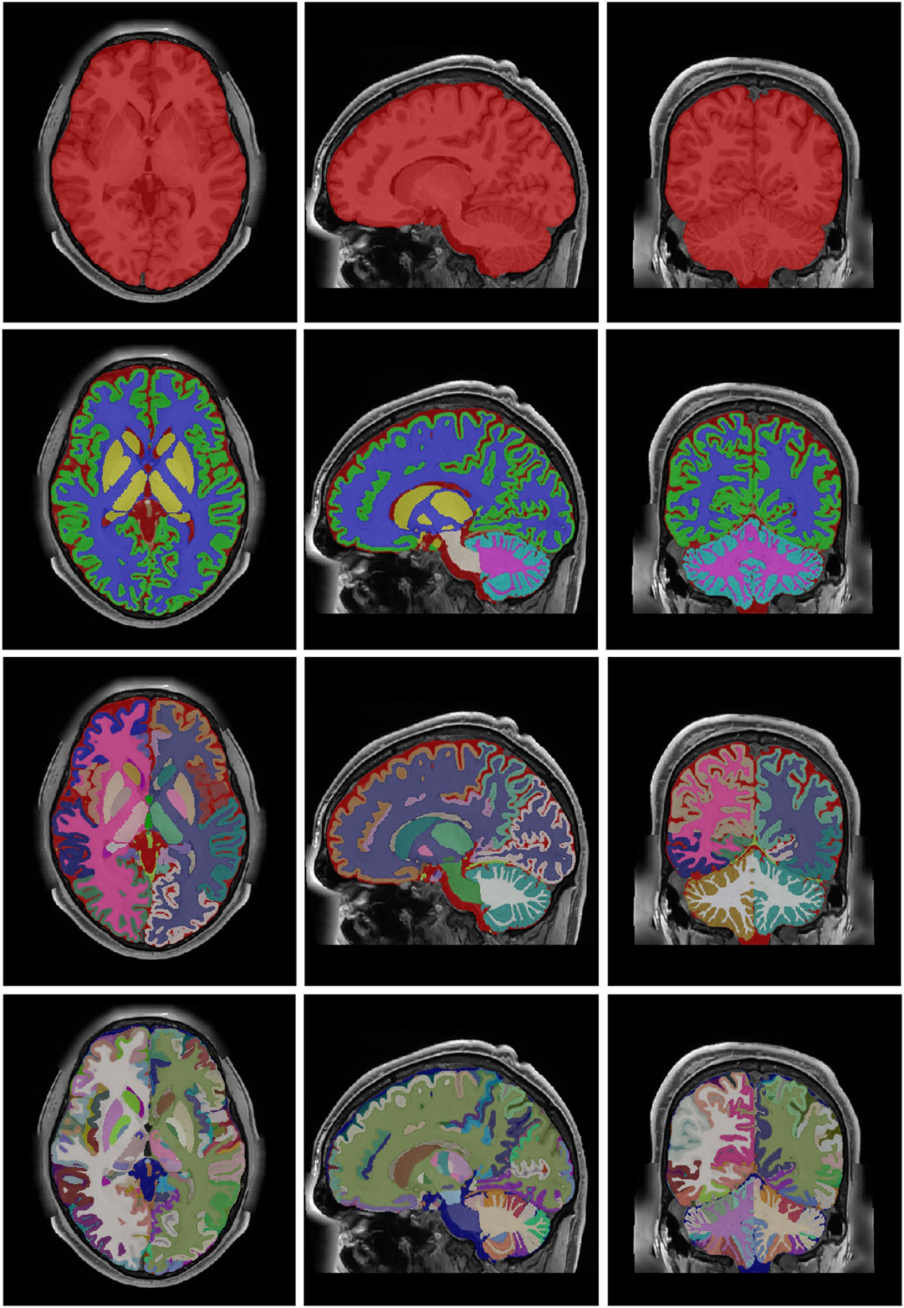
From Top to bottom: ICV (1 label), tissues (9 labels), structures (54 labels) and substructures (350 labels).

After the whole integration process, we generate the corresponding label maps at multiple scales combining the corresponding labels (from substructure to structure, then to tissue and finally to organ (ICV). Figure 11 shows an example of the new holiBrain protocol. The substructure scale has 350 labels, the structure scale has 54 labels, the tissue scale has 9 labels and finally ICV has 1 label.

### Atlas construction

To build the atlas, the 75 T1w, T2w and WMn MRI at 0.125 mm^3^ resolution at MNI152 space and their corresponding labels were used. The initial reference image used for the template generation was the MNI152 2009b Nonlinear symmetric template interpolated at 0.125 mm^3^ resolution. To create the T1w/T2w/WMn MRI templates, an iterative approach was employed. At each iteration, the 75 subjects underwent non-linear registration to the reference using the FireANTS software^50^ to estimate the non-linear deformation field. To refine the reference image, the registered images were averaged, generating a new reference image at each step. Compared to ANTs^30^ software, FireANTS provided an acceleration of 300x thanks to its robust and optimized GPU-based nature which resulted in several hours of processing compared to days using ANTS.

**Fig. 12:**
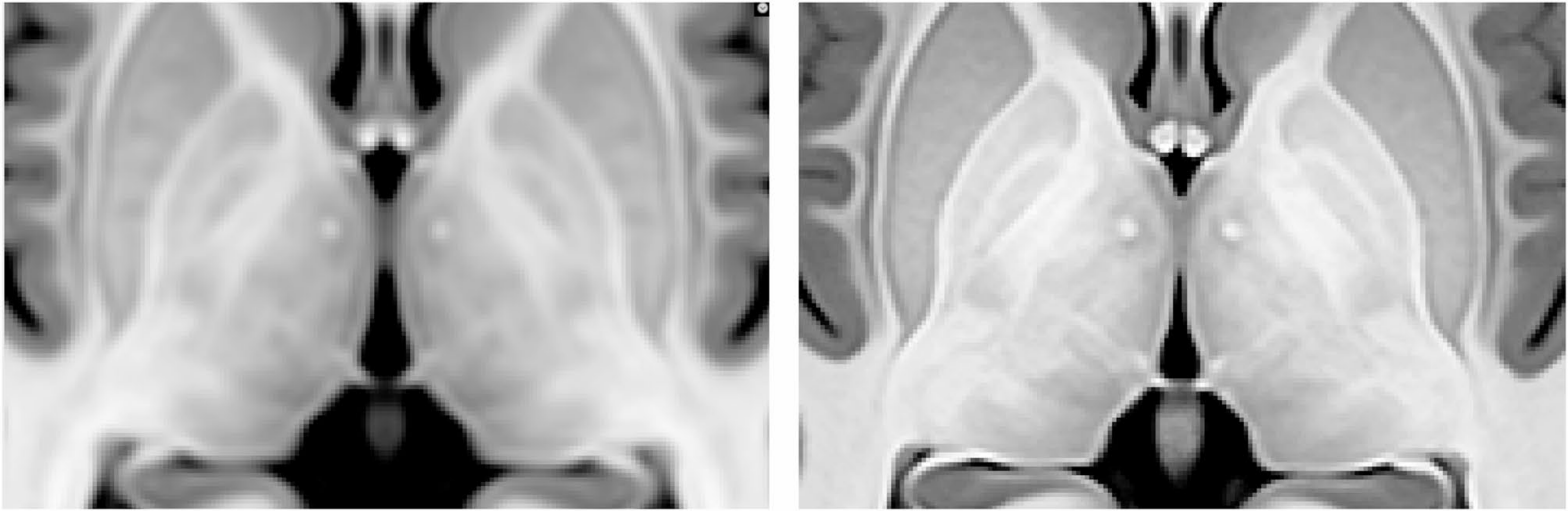
Left: Central area of the upsampled MNI152 atlas. Right: Central area of the proposed atlas. Note that the proposed atlas has a better image quality compared to classic MNI152 atlas (for example, thalamic nuclei can be better observed at the new atlas).

In total, 10 Iterations were performed for the template generation process assuring convergence. The template generation scripts used in this process are publicly available in the FireANTS repository (https://github.com/rohitrango/FireANTs). The deformation fields obtained from the MRI template construction were subsequently employed for the generation of the average T1w, T2w and WMn MRI templates, as well as for the creation of the atlas labels. T1w, T2w and WMn atlases were obtained using the median of the Laplacian-based sharpened images, which reduces blurring of the atlases (this is especially useful for small and low-contrast regions where registration might not be fully accurate). A majority voting scheme was applied to all the registered label images to estimate the atlas labels. Figure 12 shows a comparison of the proposed T1w atlas and the upsampled MNI152 T1w atlas to highlight the improved resolution of the proposed atlas.

## Results

**Fig. 13:**
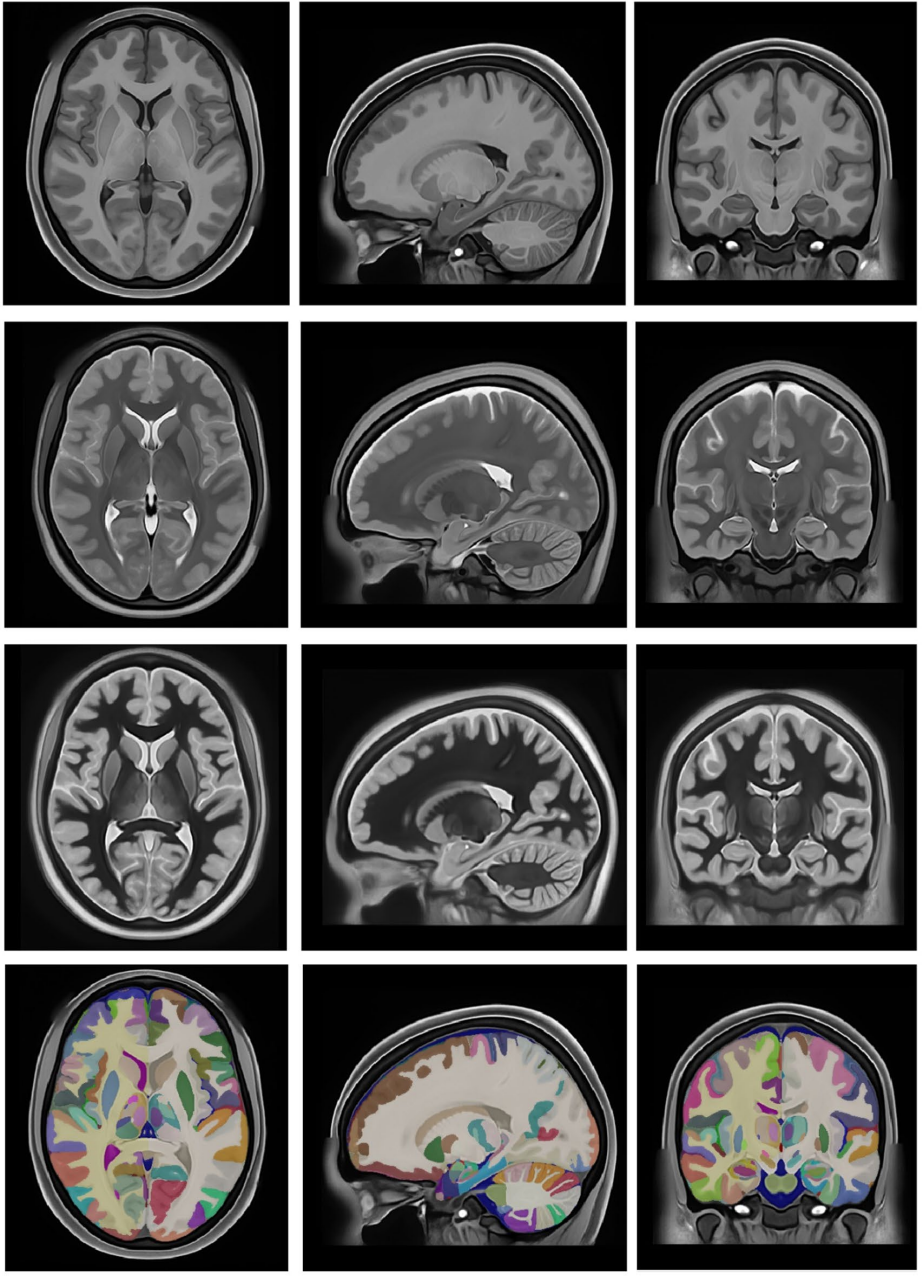
From Top to bottom: Average T1w MRI template, average T2w MRI template, average WMn MRI template and majority voting atlas labels at substructure resolution.

After the whole integration process, we generated the last version at multiple scales (substructure/structure/tissue/organ). Figure 13 shows the holiAtlas templates for T1w, T2w and WMn MRI and the corresponding labels. Label definitions and relations among scales can be inspected in the appendix. The generated holistic atlas, together with the multiscale label definitions, is publicly available through the following links: https://volbrain.net/public/data/holiatlas_v1.0.zip and https://zenodo.org/records/15690524 under a Creative Commons license.

## Discussion

This paper introduces holiAtlas, a comprehensive, multimodal, and ultra-high-resolution MRI-based atlas of the human brain anatomy. This atlas was constructed by fusing data from various local protocols with corresponding scales, resulting in a densely labelled protocol. The creation involved the use of images and segmentations from 75 healthy subjects, employing T1w, T2w, and WMn MRI contrasts at a 0.125 mm³ resolution. This atlas provides a holistic view of brain anatomy at multiple levels and scales, serving as a valuable resource for segmentation methods, research, and education.

The proposed atlas surpasses most of the existing MRI-based brain atlases in resolution, providing detailed anatomical information. Conventional atlases, like the MNI152 Atlas, offer resolutions up to 1 mm³^6^, while holiAtlas achieves a significant leap to 0.125 mm³. This finer resolution enhances the precision of structural mapping and allows for a more specific exploration of brain architecture.

holiAtlas’s unique contribution lies in the integration of diverse delineation protocols from 7 software packages. This integration process includes correction steps, such as tissue error correction, to refine and enhance the accuracy of the labels. This approach not only synthesizes information from different sources but also rectifies systematic errors, providing a more reliable atlas. During atlas integration, label overlaps were resolved using structure specific predefined anatomical priority and consistency rules, preventing overwriting inconsistent structures. This hierarchical strategy ensured consistency and reproducibility of the final segmentation.

The multimodal nature of the atlas, encompassing T1w, T2w, and WMn MRI contrasts, broadens its descriptive power as it offers a multiple view of the same organ. The multiscale holiAtlas label generation facilitates versatile usage, accommodating multiple research needs, from substructure to overall organ analysis.

Incorporating image synthesis techniques, such as generating WMn-like images using data from HCP1200, showcases the paper’s innovative approach to overcoming data limitations. This technique expands the usability of the atlas by providing a complete set of contrasts even when not all contrasts are available in the original datasets. In the future, we will add other synthetic modalities such as FLAIR or contrast-enhanced T1.

During the creation of the atlas, which took nearly 3 years and employed the expertise of many people, we emphasized the importance of manual correction and quality control in the integration process. While automated methods are powerful, the inclusion of human expertise ensures the accuracy and reliability of the final atlas. The main issue was to fuse partial (e.g., focused on specific areas or defined at a specific scale) and incompatible (e.g., a given voxel can be assigned to different structures according to the used methods) protocols into a consistent and coherent protocol across scales to obtain the final holiBrain protocol. This iterative correction process enhances the atlas validity for a broad range of applications.

Unlike other atlases, the proposed atlas is based on what one can actually see and measure in real in-vivo MR images, rather than using histological images that, despite offering impressive spatial resolution, do not match acquired MR images directly. Compared with recent high-resolution atlases^51,52^, which rely on 7 T MRI and emphasize ultra-detailed subcortical parcellations, our atlas is based on 3 T data mapping the entire brain. This makes it directly applicable to conventional research and clinical studies, offering a population-based reference for the whole that complements existing ultra–high-field resources.

We believe that the increased resolution and label density of the proposed atlas may enable earlier detection of neurological diseases by focusing on substructural atrophies rather than structure atrophy which may be more difficult to detect. For instance, the characteristic atrophy of the amygdala and the hippocampus in Alzheimer’s disease^53,54^ is due mainly to the volume reduction of the lateral and cortical amygdaloid nuclei^55^ and to neurodegeneration in the CA1 subfield^56,57^; therefore, analysis at the substructure level promises to be beneficial. A similar situation is observed in Parkinson’s disease, where volume reductions in the hippocampus and putamen are prevalent^58^ and the various clinical presentations of frontotemporal dementia^3^. In summary, the high resolution and increased label density of the present atlas will improve the analysis of individual cases at the substructural level.

The proposed atlas may play a central role in structural MRI analysis, serving as a foundational resource across a wide range of neuroimaging applications. Atlases are essential in disease mapping, acting as common coordinate systems that enable voxel-wise comparisons between populations, thereby facilitating the identification of disease-specific patterns in disorders such as Alzheimer’s disease, multiple sclerosis, or brain tumors. Moreover, longitudinal studies rely on atlases to provide consistent spatial references over time, allowing accurate tracking of brain changes due to development, aging, or neurodegeneration. In the context of machine learning, atlases serve as powerful tools for weakly supervised learning, self-supervised representation learning, and template-based data augmentation. Finally, they support the creation of population-level brain maps, helping researchers model structural variability across demographics and build normative anatomical models (similar to AAL atlas).

We acknowledge the limitations of the proposed atlas. The fact that it is based on the fusion of automatic segmentations may raise doubts about its accuracy (despite our exhaustive QC process). However, manual segmentations also have limitations. Systematic and random errors can be present in each case. Besides, modern medical image analysis software has an accuracy close to the inter-rater one which somehow justifies the use of this approach^46,59^. Even in the case of systematic errors, if these errors systematically over or underestimate the volume of interest for healthy and diseased brains in the same manner, the effect size induced by the disease can be effectively captured^60^. Furthermore, the data comes from a young cohort and may therefore not be representative of pediatric or elderly populations. Finally, while the ultra-high resolution of this atlas may not substantially improve the analysis of large structures, we expect that it will be beneficial for the study of small structures (e.g., thalamic nuclei). Besides, we are aware that the proposed atlas provides a representative population mean and it does not retain inter-individual variability, which is particularly relevant in cortical regions. This averaging may reduce sensitivity to subject-specific features; however, it offers a standardized reference space for group-level analyses.

## Conclusion

In conclusion, the holiAtlas presented in this paper stands as a novel contribution to the field of neuroscientific research and brain imaging. The integration of multiple protocols, ultra-high resolution, and multiple modalities make it a unique and valuable resource. The atlas construction methodology, combining automated segmentation with meticulous manual correction, ensures a high level of accuracy. We have addressed many limitations of existing atlases, providing a comprehensive tool that can drive advancements in segmentation methods, facilitate diverse research endeavors, and serve as an educational reference. The multimodal, multiscale, and ultra-high-resolution features of holiAtlas position it as a significant asset for the neuroscience community, offering new avenues for understanding the intricate complexities of the human brain.

## Data Availability

The generated holistic atlas, together with the multiscale label definitions, is publicly available through the following links: https://volbrain.net/public/data/holiatlas_v1.0.zip and https://zenodo.org/records/15690524 under a Creative Commons license.
